# Simple Genetic Distance-Optimized Field Deployments for Clonal Seed Orchards Based on Microsatellite Markers: As a Case of Chinese Pine Seed Orchard

**DOI:** 10.1371/journal.pone.0157646

**Published:** 2016-06-16

**Authors:** Huwei Yuan, Shihui Niu, Yousry A. El-Kassaby, Yue Li, Wei Li

**Affiliations:** 1 National Engineering Laboratory for Forest Tree Breeding, Key Laboratory for Genetics and Breeding of Forest Trees and Ornamental Plants of Ministry of Education, College of Biological Sciences and Technology, Beijing Forestry University, Beijing, China; 2 Department of Forest and Conservation Sciences, Faculty of Forestry, University of British Columbia, Vancouver, British Columbia, Canada; Wuhan Botanical Garden of Chinese Academy of Sciences, CHINA

## Abstract

Chinese pine seed orchards are in a period of transition from first-generation to advanced-generations. How to effectively select populations for second-generation seed orchards and significantly increase genetic gain through rational deployment have become major issues. In this study, we examined open- and control-pollinated progeny of the first-generation Chinese pine seed orchards in Zhengning (Gansu Province, China) and Xixian (Shanxi Province, China) to address issues related to phenotypic selection for high volume growth, genetic diversity analysis and genetic distance-based phylogenetic analysis of the selections by simple sequence repeats (SSRs), and phylogenetic relationship-based field deployment for advanced-generation orchards. In total, 40, 28, 20, and 13 superior individuals were selected from the large-scale no-pedigree open-pollinated progeny of Zhengning (ZN-NP), open-pollinated families of Zhengning (ZN-OP), open-pollinated families of Xixian (XX-OP), and control-pollinated families of Xixian, with mean volume dominance ratios of 0.83, 0.15, 0.25, and 0.20, respectively. Phylogenetic relationship analysis of the ZN-NP and XX-OP populations showed that the 40 superior individuals in the ZN-NP selected population belonged to 23 families and could be further divided into five phylogenetic groups, and that families in the same group were closely related. Similarly, 20 families in the XX-OP population were related to varying degrees. Based on these results, we found that second-generation Chinese pine seed orchards in Zhengning and Xixian should adopt a grouped, unbalanced, complete, fixed block design and an unbalanced, incomplete, fixed block design, respectively. This study will provide practical references for applying molecular markers to establishing advanced-generation seed orchards.

## Introduction

Selection and deployment of improved materials are important in establishing an advanced-generation seed orchard. To increase genetic gain, advanced-generation seed orchards usually contain only a few elite clones. To decrease the risks of inbreeding depression [1, 2], propagation populations in advanced-generation seed orchards are often composed of elite individuals from elite, pedigreed families selected through controlled pollination. However, controlled pollination is a time, resource- and cost-intensive activity, and the number of tested families is always limited, which greatly restricts the genetic quality of the selected germplasm. Open-pollinated progenies contain all possible crosses and greater numbers of superior individuals; however, such individuals are rarely used when establishing an advanced-generation seed orchard because they are not pedigreed.

Molecular markers developed in the 1980s have brought phylogenetic analysis to a new level [3]. Microsatellite markers and relevant analytical softwares have made marker-based phylogenetic analysis a reality [4, 5], and are widely used in phylogenetic analysis of natural and hybrid populations [5–8]. El-Kassaby introduced a breeding strategy called “breeding without breeding” (BWB), which has been proven highly convenient for tree breeding [9]. The efficiency of this strategy has been evaluated by progeny testing, parental selection, and construction of pedigrees [9–11]. Later, the BWB strategy was demonstrated in a number of tree species [12–14] and it has been extensively used in different areas of forest tree breeding, including phylogenetic analysis [15, 16], mating systems [12, 17–19], estimation of genetic parameters and breeding value [13, 20], and spatial variation [17]. Selecting superior individuals directly from the open-pollinated progeny of a seed orchard or from plantations established using seed orchard seeds, coupled with identifying the phylogenetic relationship of the selected materials based on molecular markers, could decrease the reliance on controlled pollination and shorten the breeding cycle by 10–15 years [9]. Genetic distance reflects the genetic relationships among materials. Simple sequence repeats (SSRs) are high-resolution markers that can identify different individuals within the same species. The combination of phenotypic selection, genetic distance-based phylogenetic analysis of selected individuals using SSR markers and phylogenetic relationship-based field deployment would simplify breeding activities, decrease inbreeding, and expand genetic diversity among seed orchard progenies.

Field deployment is one of the most important activities in seed orchard establishment. The most important criteria of field deployment is to maximize genetic gain of target traits while maintaining an acceptable level of genetic diversity [21]. Advanced-generation seed orchards of conifers often contain a small number of clones with varying origin (including backward and forward selections) [22], which has increased the probability of inbreeding and enhanced the complexity of deployment. A number of designs, including permutated neighborhood design [23], systematic layout [22], randomized, replicated, staggered clonal-row (R^2^SCR) design [24], have been employed in the deployment of advanced-generation clonal seed orchards. It was proved that unequal clonal deployment could improve genetic gain at a certain level of genetic diversity [21, 25, 26], and the mating system of advanced-generation clonal seed orchards could be controled by allocating clones based on their phylogenetic relationships [27].

Chinese pine (*Pinus tabuliformis*) is a major afforestation tree species that is naturally distributed in the 3,000,000 km^2^ of mountainous areas between 32°–43°N and 102°–122°E in northern China [28–30]. Genetic improvement of Chinese pine was initiated in the 1970s [31]. Sixteen first-generation seed orchards containing more than 4,000 plus-tree clones were established, and about 1,000 open-pollinated and 200 control-pollinated families were tested [32]. Variations in flowering and fruiting characteristics among clones in the seed orchards were studied extensively [33–38] and improved seeds from the orchards were used for afforestation. Chinese pine seed orchards are currently in a period of transition from first-generation to advanced-generation orchards; thus, how to select superior individuals from the available material and form rational deployment designs of clones in advanced-generation seed orchards have become major concerns for breeders.

In this study, we examined large-scale no-pedigree open-pollinated progeny, and open- and control-pollinated families of the first-generation Chinese pine seed orchards in Zhengning and Xixian as a base population. Our aims were to: screen polymorphic SSR primers adapted to Chinese pine, pre-select superior individuals in the base population based on volume growth, identify the relative phylogenetic relationships among the selected individuals, and develop a simplified clone deployment strategy for the corresponding advanced-generation seed orchards. Our results will provide practical references for applying molecular markers in Chinese pine improvement programs.

## Materials and Methods

### Screening of polymorphic SSR primers for Chinese pine

We extracted genomic DNA from the needles of Chinese pine using the CTAB method [39]. Ours is the leading cooperative group researching Chinese pine, and comprises the Zhengning Forestry Institute in Gansu Province and the Lüliang Mountain State-owned Forest Administration in Shanxi Province. Both authorities gave full permission to conduct all studies at the experimental sites. This study did not involve endangered or protected species. Twenty-one SSR primers from Chinese pine and related species [40] were synthesized by Sangon Biotech Co. Ltd. (Beijing, China) and used for polymerase chain reaction (PCR) amplification. PCR amplification was performed on a Veriti Dx 96-well Thermal Cycler (Applied Biosystems, Foster City, CA, USA) using a method described previously [41]. The amplified PCR fragments were separated on 8% polyacrylamide gels using an HT-SCZ04 Vertical Electrophoresis Tank (Hongtaojiye Science & Technology Co. Ltd., Beijing, China) according to the manufacturer’s instructions. Primers that could present polymorphic amplification products among individuals were screened and used for the following experiment. Detailed information regarding the 11 polymorphic SSR primers is shown in Table 1.

**Table 1:** Characteristics of the *P*. *tabuliformis* polymorphic simple sequence repeat (SSR) primers.

Primer No.	Locus	Primer sequences (5′-3′)	Expected size (bp)	T_a_ (°C)
**1**	LOP1	F: GGCTAATGGCCGGCCAGTGCT	158	58
**1**	LOP1	R: GCGATTACAGGGTTGCAGCCT	158	58
**2**	PtTX2146	F: CCTGGGGATTTGGATTGGGTATTTG	200	51
**2**	PtTX2146	R: ATATTTTCCTTGCCCCTTCCAGACA	200	51
**3**	PtTX3107	F: AAACAAGCCCACATCGTCAATC	160	54
**3**	PtTX3107	R: TCCCCTGGATCTGAGGA	160	54
**4**	PtTX3116	F: CCTCCCAAAGCCTAAAGAAT	160	54
**4**	PtTX3116	R: CATACAAGGCCTTATCTTACAGAA	160	54
**5**	PtTX4001	F: CTATTTGAGTTAAGAAGGGAGTC	210	52
**5**	PtTX4001	R: CTGTGGGTAGCATCATC	210	52
**6**	SPAC12.5	F: CTTCTTCACTAGTTTCCTTTGG	130	56
**6**	SPAC12.5	R: TTGGTTATAGGCATAGATTGC	130	56
**7**	ctg1367	F: CGATATTATGGATTTTGCTTGTGA	120	53
**7**	ctg1367	R: AAATGCATGCCAAACTTAAATAC	120	53
**8**	pdms221	F: GAGAGTTGTATGACGGAAATAC	165	54
**8**	pdms221	R: CCCACACAAAAGTGTACTTC	165	54
**9**	CR354677	F: ATGGTGGTTGATCTGCAGGCTAAT	230	51
**9**	CR354677	R:GTTTAACCAACCGCACTCATTTTTCACT	230	51
**10**	PtTX2123	F: GAAGAACCCACAAACACAAG	210	57
**10**	PtTX2123	R: GGGCAAGAATTCAATGATAA	210	57
**11**	RPS160	F: ACTAAG AACTCTCCCTCTCACC	240	55
**11**	RPS160	R: TCATTGTTCCCCAAATCAT	240	55

Note: T_a_, annealing temperature.

### Base population and selection of superior individuals

The first-generation Chinese pine seed orchards in Zhengning (Gansu Province) and Xixian (Shanxi Province) contained about 300 clones each. We regarded no-pedigree open-pollinated progeny of clones in the Zhengning seed orchard (ZN-NP), open-pollinated families of clones in the Zhengning seed orchard (ZN-OP), open-pollinated families of clones in the Xixian seed orchard (XX-OP), and control-pollinated families of clones in the Xixian seed orchard (XX-CP) as the base population, with ages of 21, 21, 22, and 22 years, respectively. We measured individual's heights and diameters at 1.3 m, and calculated individual's volume as:

$$V=2\pi \cdot \left(D_{1.3}/2\right)\times \left(H+3\right)\times f$$

where *V* is the individual's volume, *f* is 0.3578 for Chinese pine, *D*_1.3_ is diameter at 1.3 m height, and *H* is height. Individuals or families in the base population were ranked according to their volume dominance ratio (*R*_d_), which was calculated as:

$$R_{\text{d}}=(V-\bar{V})/\bar{V}$$

where *V* is an individual tree's volume or a family's mean volume; and $\bar{V}$ is the mean volume of the corresponding population. The selected population in ZN-NP was composed of individuals with high-ranked *R*_d_ values in the base population; the ZN-OP, XX-OP and XX-CP selected populations were composed of high-ranked individuals (individuals with high R_d_ values) within the high-ranked families (families with high R_d_ values) in the corresponding base populations. We conducted analyses of variance (ANOVAs) and multiple comparisons using R 2.15.3 software to describe differences in *R*_d_ values among the selected populations.

### Genetic distance-based phylogenetic analysis and genetic diversity analysis of superior individuals in the ZN-NP and XX-OP selected populations

We used superior individuals in the ZN-NP and XX-OP selected populations as samples for the phylogenetic analysis. We performed DNA extraction, PCR amplification, and polyacrylamide gel electrophoresis using the methods described above, and analyzed the genotyping results using PowerMarker 3.25 software. We also calculated frequency and frequency-based distance (Nei 1983), and reconstructed genetic distance-based phylogenetic trees using the unweighted pair-group method with the arithmetic average method. Phylogenetic trees were viewed using Fig.Tree 1.4.2 software.

We analyzed the genetic diversity of superior individuals in the ZN-NP and XX-OP selection populations using PowerMarker 3.25 software. The parameters analyzed included observed heterozygosity (*H*_o_), expected heterozygosity (*H*_e_), and polymorphism information content (*PIC*).

## Results

### Selected populations in the second-generation Chinese pine seed orchards

**Fig 1:**
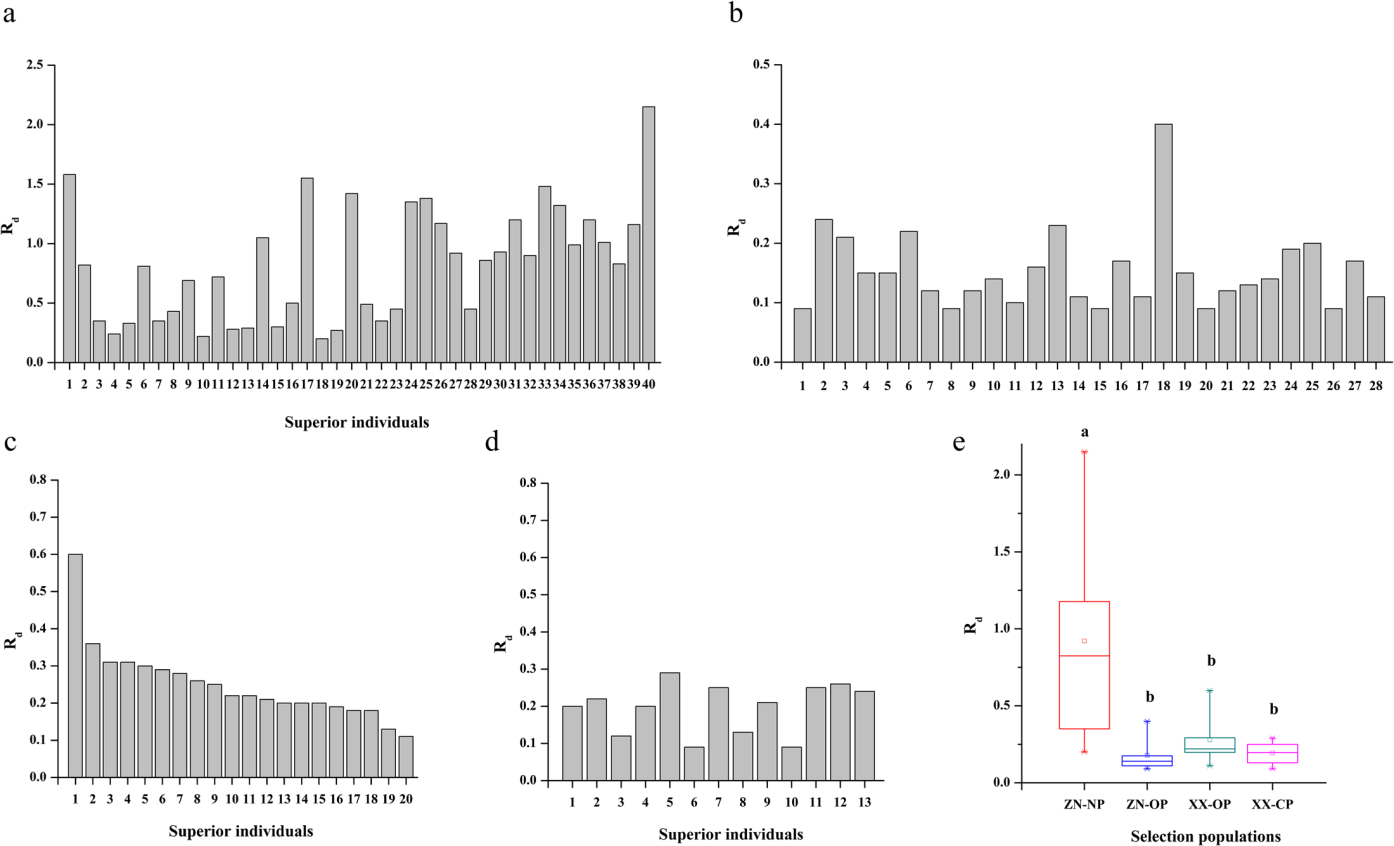
Volume dominance ratios (R_d_) of the second-generation selected populations of Chinese pine. Comments: (a) Volume dominance ratios of superior individuals in the ZN-NP selected population; (b) volume dominance ratios of superior individuals in the ZN-OP selected population; (c) volume dominance ratios of superior individuals in the XX-OP selected population; (d) volume dominance ratios of superior individuals in the XX-CP selected population; (e) Variations in volume dominance ratios among the four selected populations.

The second-generation selected populations consisted of superior individuals with high-ranking *R*_d_ values. The ZN-NP, ZN-OP, XX-OP, and XX-CP selected populations had *R*_d_ values of 0.20–2.15, 0.09–0.40, 0.11–0.60, and 0.09–0.29, with mean values of 0.83, 0.15, 0.25, and 0.20, respectively (Fig 1a–1d). ANOVA showed that *R*_d_ values were significantly higher in the ZN-NP selected population than in the other selected populations, whereas there were no statistically significant differences in *R*_d_ values among the ZN-OP, XX-OP, and XX-CP selected populations (Fig 1e).

### Genetic distance-based phylogenetic analysis of superior individuals in the ZN-NP and XX-OP selected populations

**Fig 2:**
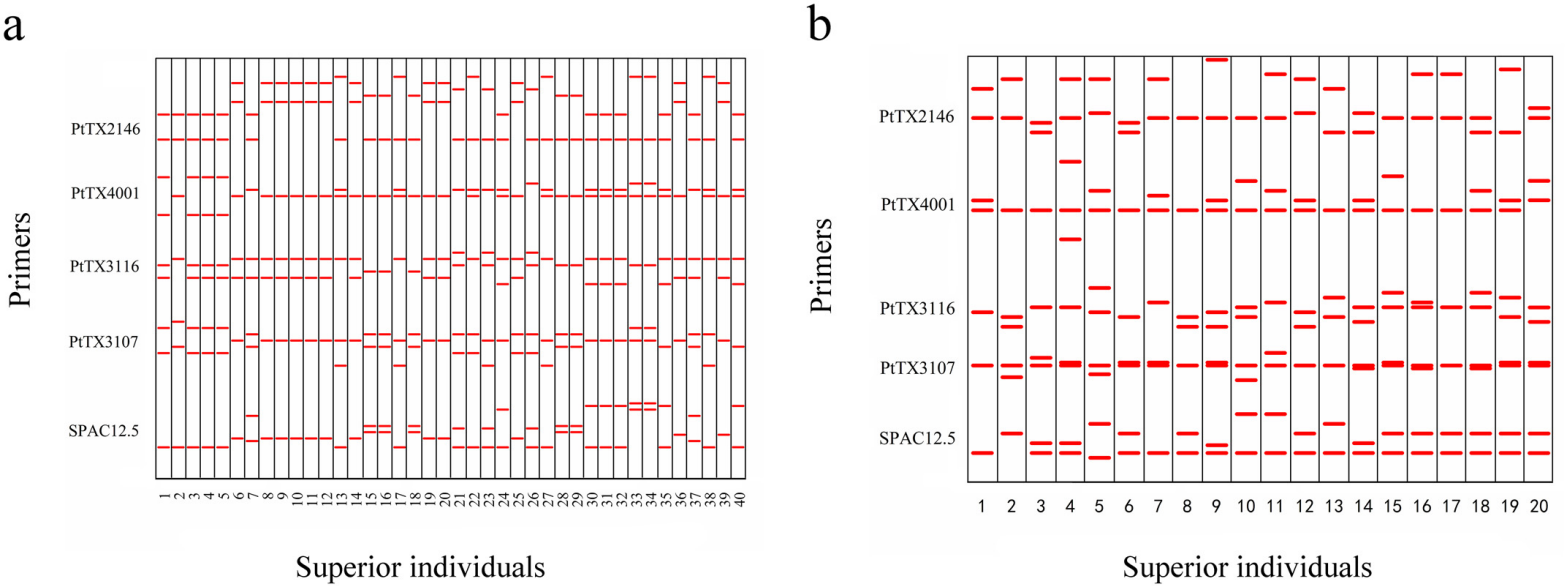
Fingerprints of five simple sequence repeat (SSR) primers in the second-generation ZN-NP (a) and XX-OP (b) selected populations of Chinese pine.

**Fig 3:**
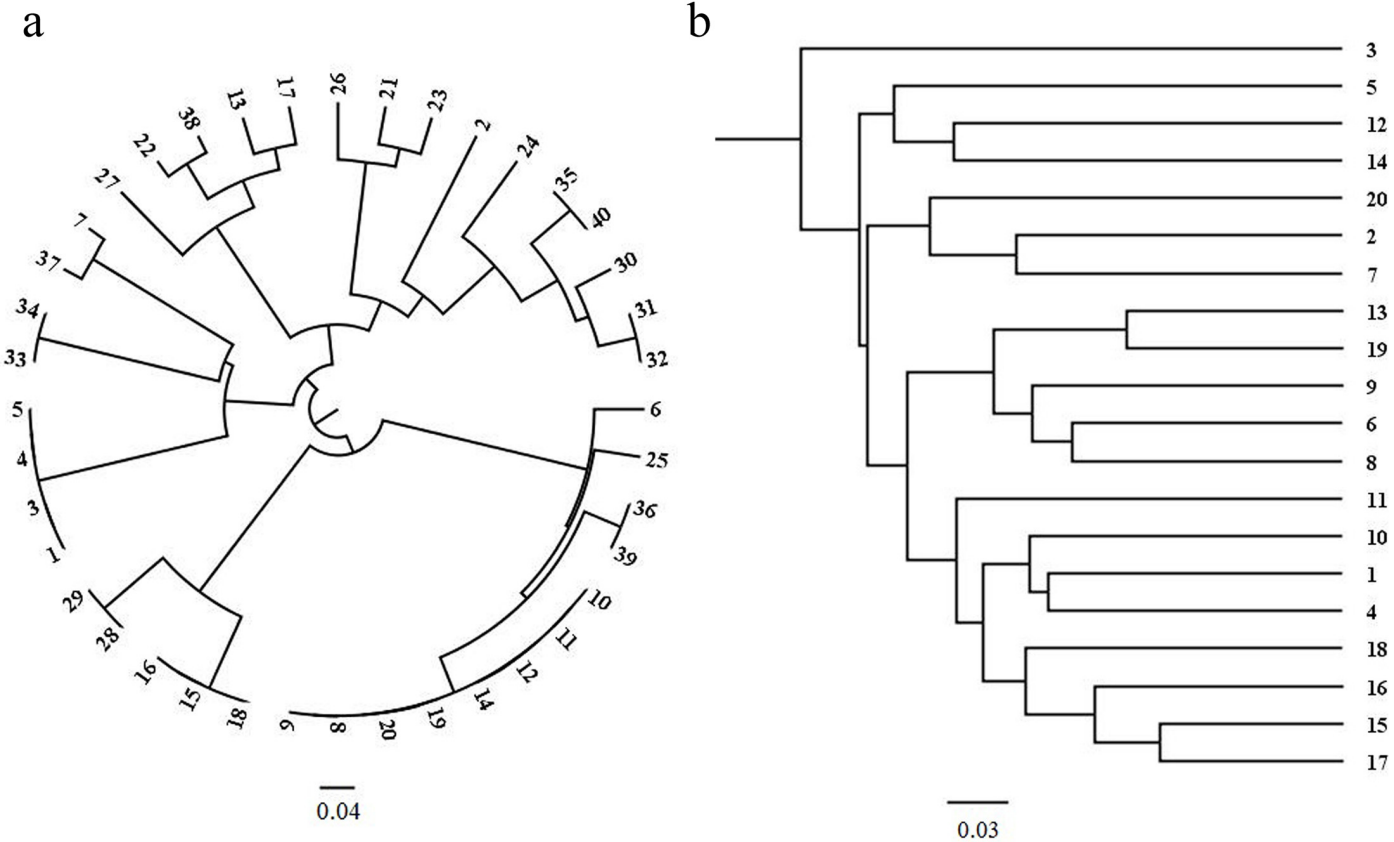
Phylogenetic trees of superior individuals in the second-generation ZN-NP (a) and XX-OP (b) selected populations of Chinese pine.

The fingerprints revealed that the amplified bands were polymorphic among individuals in the ZN-NP and XX-OP selected populations (Fig 2). The phylogenetic analysis revealed that the 40 individuals in the ZN-NP selected population belonged to 23 families with various numbers of individuals. The largest family consisted of eight individuals (nos. 8, 9, 10, 11, 12, 14, 19, and 20). The numbers of families containing one, two, three, and four individuals were 15, 5, 1, and 1, respectively (Fig 3a). We found a close relationship between the family consisting of individuals 28 and 29 and the family consisting of individuals 15, 16, and 18, whereas the relationships between these two families and the other families were distant. Similarly, families consisting of individuals 13, 17, 22, 27, and 38 were closely related but they were distantly related to the other families. The details of the phylogenetic relationships among the families in the ZN-NP selected population are shown in Fig 3a.

The 20 individuals in the XX-OP selected population belonged to 20 different families (Fig 3b). The families were numbered the same as the individuals. Family 3 was distantly related to the other families. Families 5, 12, and 14 were closely related but were distantly related to the other families. Families 2, 7, and 20 were closely related but were distantly related to the other families. The details of the phylogenetic relationships among families in the XX-OP selected population are shown in Fig 3b.

### Genetic diversity of superior individuals in the ZN-NP and XX-OP selected populations

Table 2 shows that the major allele frequency (*MAF*), the number of genotypes (No. genotypes), and the number of alleles (No. alleles) of the 11 polymorphic SSR loci in the ZN-NP selected population had ranges of 0.3250–0.6875, 4.0000–10.0000, and 3.0000–11.0000, with means of 0.4568, 6.0909, and 5.5455, respectively. Expected heterozygosity (*H*_*e*_) and observed heterozygosity (*H*_*o*_) values varied from 0.4609 to 0.7931 and from 0.1000 to 1.000, respectively, with means of 0.6690 and 0.5614. Polymorphism information content (*PIC*) changed from 0.3977 to 0.7671, with a mean of 0.6202. The fixation index (*F*) had a range of −0.2676–0.8532, with a mean of 0.1732.

**Table 2:** Genetic diversity in the selected second-generation Chinese pine ZN-NP population.

Primer No.	*MAF*	No. genotype	No. allele	*H*_*e*_	*H*_*o*_	*PIC*	*F*
**1**	0.3250	10.0000	7.0000	0.7663	0.9000	0.7314	-0.1623
**2**	0.3500	5.0000	7.0000	0.7931	1.0000	0.7671	-0.2490
**3**	0.4500	9.0000	7.0000	0.7328	0.5750	0.7036	0.2274
**4**	0.4000	7.0000	6.0000	0.7416	0.6750	0.7042	0.1023
**5**	0.6125	6.0000	5.0000	0.5609	0.4250	0.5121	0.2542
**6**	0.3625	9.0000	11.0000	0.7872	0.4500	0.7631	0.4386
**7**	0.5750	4.0000	5.0000	0.5863	0.7500	0.5301	-0.2676
**8**	0.6875	4.0000	3.0000	0.4609	0.3250	0.3977	0.3064
**9**	0.3625	5.0000	3.0000	0.6653	0.1000	0.5913	0.8532
**10**	0.4000	4.0000	4.0000	0.6550	0.3000	0.5877	0.5509
**11**	0.5000	4.0000	3.0000	0.6097	0.6750	0.5335	-0.0946
**Mean**	0.4568	6.0909	5.5455	0.6690	0.5614	0.6202	0.1732

Comments: *MAF*, *H*_*e*_, *H*_*o*_, *PIC*, and *F* are major allele frequency, expected heterozygosity, observed heterozygosity, polymorphism information content, and the fixation index, respectively.

Table 3 shows that the *MAF*, numbers of genotypes, and numbers of alleles of the 11 polymorphic SSR loci in the selected XX-OP population had ranges of 0.2750−0.9500, 2.0000−13.0000, and 2.0000−10.0000, with means of 0.5846, 7.1818, and 6.0000, respectively. *H*_*e*_ and *H*_*o*_ varied from 0.0950 to 0.8438 and from 0.1000 to 0.8500, respectively, with means of 0.5703 and 0.5955. The *PIC* changed from 0.0905 to 0.8266, with a mean of 0.5324. *F* ranged between −0.2925 and 0.3956, with a mean of −0.0184.

**Table 3:** Genetic diversity in the second-generation XX-OP selected population of Chinese pine.

Primer No.	*MAF*	No. genotype	No. allele	*H*_*e*_	*H*_*o*_	*PIC*	*F*
**1**	0.6000	10.0000	7.0000	0.6013	0.6000	0.5725	0.0277
**2**	0.4000	12.0000	10.0000	0.7838	0.8500	0.7634	-0.0590
**3**	0.6250	8.0000	8.0000	0.5700	0.7500	0.5387	-0.2925
**4**	0.2750	13.0000	10.0000	0.8438	0.7000	0.8266	0.1952
**5**	0.6500	8.0000	7.0000	0.5450	0.6500	0.5170	-0.1678
**6**	0.5000	8.0000	7.0000	0.6625	0.8500	0.6169	-0.2593
**7**	0.9500	2.0000	2.0000	0.0950	0.1000	0.0905	-0.0270
**8**	0.7000	3.0000	3.0000	0.4338	0.5500	0.3589	-0.2440
**9**	0.5500	8.0000	6.0000	0.6388	0.4000	0.6015	0.3956
**10**	0.5500	3.0000	3.0000	0.5950	0.5000	0.5280	0.1845
**11**	0.6500	4.0000	3.0000	0.5050	0.6000	0.4425	-0.1633
**Mean**	0.5864	7.1818	6.0000	0.5703	0.5955	0.5324	-0.0184

Comments: *MAF*, *H*_*e*_, *H*_*o*_, *PIC* and *F* are major allele frequency, expected heterozygosity, observed heterozygosity, polymorphism information content and the fixation index, respectively.

### Deployment designs for the second-generation Chinese pine clonal seed orchards in Zhengning and Xixian

We conducted secondary selection among the elementarily selected individuals in the ZN-NP and XX-OP selected populations before preparing the deployment designs. Families with ≥ 2 individuals underwent within-family selection and only individuals with the highest *R*_d_ values were selected in the corresponding families. Designs were prepared based on the criteria of maintaining maximum genetic distance and genetic diversity, increasing the frequency of the best selections, and fixed deployment.

**Fig 4:**
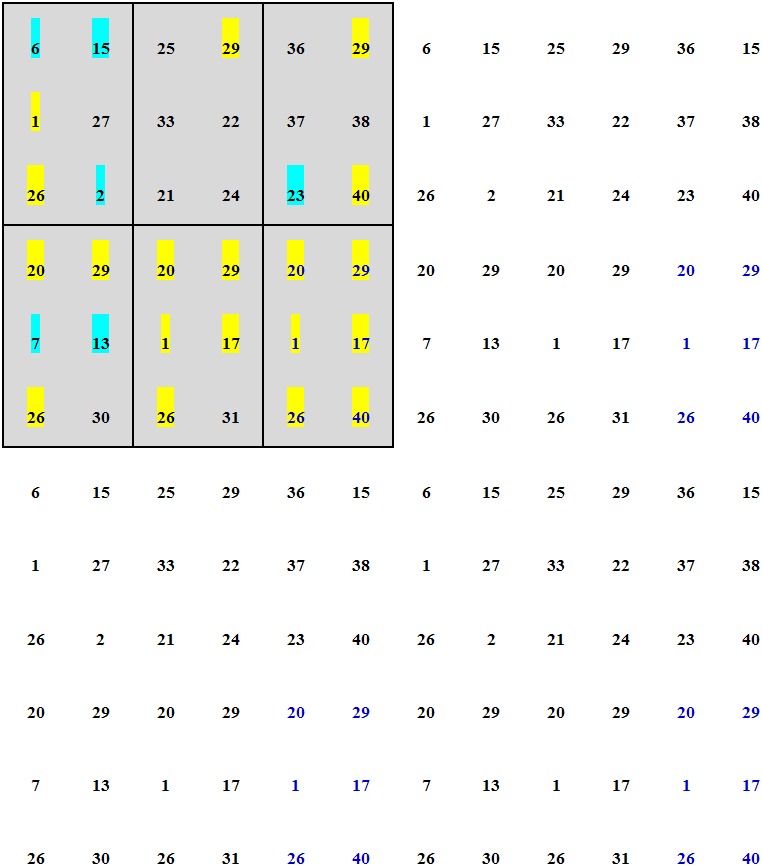
Field deployment for the second-generation Chinese pine seed orchard in Zhengning. Note: Number represents family no. Families in the same rectangle belong to the same deployment group (G_d_). Families in blue belong to the G_d_ added. Families with yellow shading, blue shading, and without shading are the best, worst, and moderate families, respectively. The grey shaded areas represent one complete block, and four blocks are shown.

Seventeen individuals were removed by secondary selection, and 23 families with only one individual each were included in the final ZN-NP selected population. The 23 families were divided into six phylogenetic groups (G_p_) according to their phylogenetic relationships (Fig 3a). Families within the same group were closely related, and families in different groups were distantly related. For example, families 6, 25, 36, and 20 were included in the same G_p_, and families 15 and 29 were in the same G_p_ (Fig 3a). The details of the grouping referred to Fig 3a. From two to five families were in different G_p_. We adopted a grouped, unbalanced, complete, fixed block (GUCFB) design for the second-generation Chinese pine seed orchards in Zhengning (Fig 4). We chose one family from each of the six G_p_, and the six selected families with a distant genetic relationship formed a deployment group (G_d_; families in the same rectangle of Fig 4 were in the same G_d_). Five G_d_ were needed when all 23 families were included. The families with the highest *R*_d_ values in each G_p_ (families 20, 29, 1, 17, 26, and 40 in blue in Fig 4) were used to complement if there were fewer than five families in the corresponding G_p_. Next, we added the G_d_ consisting of the families with the highest *R*_d_ values in each G_p_ (the rectangle with family no. in blue in Fig 4). Finally, six G_d_ were included in the design and formed a block (areas with grey shading belonged to a complete block). All blocks were the same in the design (fixed design). Within each block, 19 (52.78%, families with yellow shading in Fig 4, 6 (16.67%, families with the blue shading in Fig 4), and 11 (30.56%, families without shading in Fig 4) best families (families with the highest *R*_d_ of volume in each G_p_), worst families (families with the lowest *R*_d_ of volume in each G_p_), and moderate families (the remaining families) were used. Consequently, the families were deployed unequally. Four blocks are shown in Fig 4.

**Fig 5:**
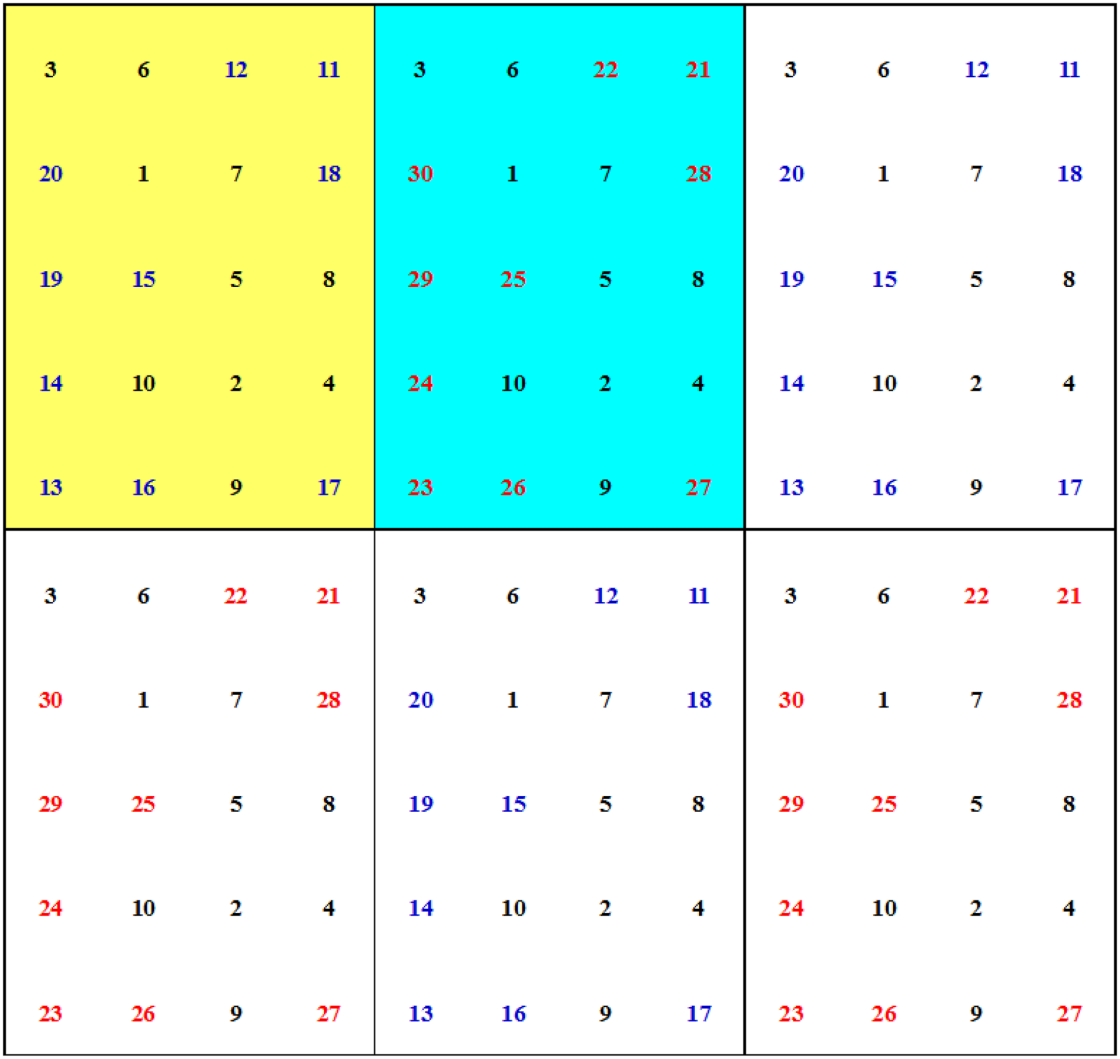
Field deployment of the second-generation Chinese pine seed orchard in Xixian. Note: Number represents family no. Families in the same rectangle belong to the same block. Families in black, blue, and red fonts are the best, moderate, and control-pollinated families. The yellow and blue shaded areas represent the two kinds of complete blocks, and six blocks are shown.

As only one individual in each family was from the XX-OP selected population, there was no need for secondary selection. The 20 families in the XX-OP selected population (nos. 1–20 in Fig 5) and the 10 families in the XX-CP selected population (top 10 families in Fig 1d; nos. 21–30 in Fig 5) with clear genetic relationships were deployed together. We adopted an unbalanced, incomplete, fixed block (UIFB) design for the second-generation Chinese pine seed orchards in Xixian. We included twenty families in each block with single-tree plot, and considered the phylogenetic relationships among families. We divided the 20 families in the XX-OP selected population into two groups. Families 1–10 were the best families (with high *R*_d_ values; families in black font in Fig 5) and families 11–20 were the moderate families (with lower *R*_d_ values; families in blue font in Fig 5). The best families were deployed in every block, and the moderate families and control-pollinated families (families in red font in Fig 5) were deployed in every other block. The frequency of the best families was twice that of the moderate families and the control-pollinated families (unequal deployment) across the entire design. Blocks containing the same families were of the same (fixed design). The two kinds of blocks (one containing families 1–20 with yellow shading in Fig 5; the other containing families 1–10 and 21–30 with blue shading in Fig 5) were alternately deployed in the adjacent blocks. Six blocks are shown in Fig 5.

## Discussion

### Strategies for selecting the second-generation selected populations of Chinese pine

Four second-generation base populations were used in this study. The ZN-NP base population consisted of bulked no-pedigree open-pollinated progeny of the first-generation Chinese pine seed orchard in Zhengning with a large planting scale. Neither of the paternal or maternal information in the ZN-NP base population were known. The ZN-OP and XX-OP base populations were composed of open-pollinated families of the first-generation Chinese pine seed orchards in Zhengning and Xixian. The maternal sources of the individuals were known but the paternal information was not. The XX-CP base population contained the control-pollinated families of the first-generation Chinese pine seed orchards in Xixian, with known parental information.

The second-generation selected populations were composed of individuals with relatively high *R*_d_ values in the corresponding base population. The mean *R*_d_ values in the selected ZN-NP, ZN-OP, XX-OP, and XX-CP populations were 0.83, 0.15, 0.25, and 0.20, respectively (Fig 1a–1d). The mean *R*_d_ value was significantly higher in the ZN-NP selected population than in the other populations (Fig 1e). This may have occurred because the ZN-NP base population comprised randomly mated progeny of all clones in the first-generation seed orchard, contained nearly all possible crosses needed for testing, and had relatively high genetic diversity and selection potential, whereas the open- and control-pollinated families contained only limited numbers of families that needed to be tested, and had relatively low levels of genetic diversity and selection potential. The mean *R*_d_ value was slightly but not significantly higher in the XX-OP selected population than in the XX-CP selected population (Fig 1e), which might be explained that although the XX-OP base population had higher number of tested families and level of genetic diversity, the XX-CP base population had better genetic basis, as all tested families were derived from pre-selected clones in the first-generation seed orchard.

In general, *R*_d_ values were significantly higher in selected populations derived from large-scale bulked no-pedigree open-pollinated progeny than in those derived from the common open- and control-pollinated progeny, indicating that elite families could be selected effectively from the large-scale open-pollinated progeny for future genetic improvement of Chinese pine, which could significantly increase the genetic gain of the target trait. In addition, we detected no differences between the open- and control-pollinated selected populations, but the control-pollination process is much more labor- and time-consuming. Thus, selecting elite families from the open-pollinated progeny could significantly shorten the breeding cycle and increase improvement efficiency.

### Effectiveness of SSR markers in the phylogenetic analysis of Chinese pine progeny

SSR molecular markers developed in the 1980s are widely used in phylogenetic analysis and population genetics because of their neutral evolution, abundant polymorphic loci, even distribution, co-dominance, stability, and repeatability [42–43]. In this study, we screened 11 polymorphic SSR primers from studies of Chinese pine and related species [40]. We used these primers to amplify stable, clear, polymorphic, and repeatable 120- to 240-bp bands in different Chinese pine individuals at annealing temperatures of 51–58°C (Table 1).

Inbreeding is a serious problem when attempting to improve tree traits because it can cause inbreeding depression and decrease the improvement effect [1, 2]. Advanced-generation seed orchards usually contain fewer clones derived from backward selection, forward selection, or both, compared with those of first-generation seed orchards [22], leading to a great increase in inbreeding rates. Consequently, the phylogenetic relationships among clones must be considered to minimize inbreeding and ensure improvement. Of the four selected populations, both maternal and paternal information was unknown for the ZN-NP population, and the paternal information was unknown for the ZN-OP and XX-OP populations. The representative ZN-NP and XX-OP selected populations were chosen for phylogenetic analysis. We used the 11 polymorphic primers screened in the previous section to identify the phylogenetic relationships among individuals in the ZN-NP and XX-OP selected populations. The amplified bands were polymorphic among individuals from the ZN-NP and XX-OP selected populations (Figs 2 and 3), with mean *PIC* values of 0.6202 (Table 2) and 0.5324 (Table 3), respectively. Families in the ZN-NP selected population had varying levels of phylogenetic relationship. Some families in the ZN-NP selected population had more than one individual, and the largest family had eight individuals (individuals 8, 9, 10, 11, 12, 14, 19, and 20) (Fig 3a), demonstrating that this family had a significant advantage in volume growth. It also reveals that there was a high probability that the same families would be selected multiple times if they were selected from the no-pedigree open-pollinated progeny, so more candidate individuals must be used to ensure a particular number of families. All individuals in the ZN-NP selected population were divided into five phylogenetic groups (G_p_), and families within the same G_p_ were closely related, whereas families in different G_p_ were distantly related. For example, the family composed of individuals 28 and 29 and the family composed of individuals 15, 16, and 18 were in the same G_p_. Similarly, individuals 13, 17, 22, 27, and 38 were in the same G_p_ (Fig 3a). The phylogenetic analysis of individuals in the XX-OP selected population showed that family 3 was distantly related to the other families. Families 5, 12, and 14, as well as families 2, 7, and 20 were closely related (Fig 3b). Although the exact parents of the individuals were not identified using the SSR molecular markers, the relative phylogenetic relationships among individuals in the selected population were revealed. Inbreeding can be avoided by deployment designs that consider the relative phylogenetic relationships among individuals and families. Consequently, SSR molecular markers could be used efficiently during marker-assisted selection and to determine the phylogenetic relationships among individuals within the Chinese pine population.

### Genetic distance-based field deployments for second-generation Chinese pine seed orchards

Seed orchards are designed and established to produce superior seeds or other products [44, 45]. The most important purpose of a seed orchard is to maximize the genetic gain of target traits while maintaining certain levels of genetic diversity. The effective population size, mating system, and flowering phenology can directly or indirectly affect genetic diversity or genetic gain and are major considerations in orchard deployment design [22]. The first-generation seed orchards in Zhengning and Xixian adopted an ordinal offset staggered design, which is feasible for first-generation seed orchards because the number of clones is relatively high and the clones are distantly related. Advanced-generation seed orchard designs are much more complex because of the limited number of clones, diverse sources of the clones (backward selection, forward selection, and so on) and higher level of inbreeding probability.

The second-generation Chinese pine seed orchards in Zhengning adopted a GUCFB design (Fig 4). The 23 families in the secondary-selected population at Zhengning were divided into six deployment groups (G_d_) according to their relative phylogenetic relationships. Each G_d_ contained six families, with one best individual each, derived from a different G_p_. Families in the same G_d_ were distantly related. In each block, the proportions of best, moderate, and worst families were 52.78%, 30.56%, and 16.67%, respectively. All 23 families were included in a block, and the blocks were the same across the entire design (Fig 4). The grouped fixed design increases the possibility of outbreeding and allows closely related progeny to be used effectively in an advanced-generation seed orchard. Following a method reported previously [25, 46], we used an unbalanced block design, which increased the mean *R*_d_ value from 1.00 in the balanced design to 1.76, thus maximizing genetic gain at the same level of genetic diversity.

Twenty open-pollinated families (Fig 1c) and 10 control-pollinated families (top 10 families in Fig 1d) were included in the second-generation seed orchard at Xixian. A UIFB design was adopted. Twenty families were included in each block. The 10 best families were deployed in every block, and the 10 moderate and 10 control-pollinated families were deployed in every other block. The relative positions were fixed (Fig 5); this is the recommended systematic design for advanced-generation seed orchards [22]. We considered the relative phylogenetic relationships among the families when determining the relative positions of the families to maintain a certain distance between related families and to control inbreeding. We also considered an unbalanced design, in which the number of best families was twice that of moderate and control-pollinated families across the entire design. This unbalanced design increased the mean *R*_d_ value from 0.24 in the balanced design to 0.34, which increased genetic gain at the same level of genetic diversity.
